# Exploring the genetic space of the DNA damage response for cancer therapy through CRISPR‐based screens

**DOI:** 10.1002/1878-0261.13272

**Published:** 2022-06-29

**Authors:** Jordan Wilson, Joanna I. Loizou

**Affiliations:** ^1^ Center for Cancer Research, Comprehensive Cancer Centre Medical University of Vienna Austria; ^2^ CeMM Research Center for Molecular Medicine of the Austrian Academy of Sciences Vienna Austria

**Keywords:** cancer therapy, CRISPR‐Cas9 screens, DNA damage response, drug discovery, synthetic lethality, synthetic viability

## Abstract

The concepts of synthetic lethality and viability have emerged as powerful approaches to identify vulnerabilities and resistances within the DNA damage response for the treatment of cancer. Historically, interactions between two genes have had a longstanding presence in genetics and have been identified through forward genetic screens that rely on the molecular basis of the characterized phenotypes, typically caused by mutations in single genes. While such complex genetic interactions between genes have been studied extensively in model organisms, they have only recently been prioritized as therapeutic strategies due to technological advancements in genetic screens. Here, we discuss synthetic lethal and viable interactions within the DNA damage response and present how CRISPR‐based genetic screens and chemical compounds have allowed for the systematic identification and targeting of such interactions for the treatment of cancer.

## Graphical abstract

The combination of CRISPR gene editing and drug treatments in a pooled screening format allows for the discovery of novel gene‐drug interactions. These interactions can potentially provide biomarkers for drug sensitivity and resistance in a clinical setting.

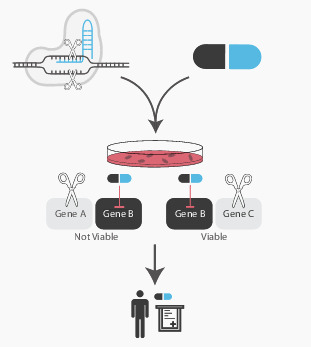

**Abbreviations**
ABE: adenine base editors
BER: base excision repair
CBE: cytosine base editors
CRISPRa: CRISPR activation
CRISPRi: CRISPR interference
Cryo‐EM: cryo‐electron microscopy (EM)
DDR: DNA damage response
FACS: fluorescence‐activated cell sorting
FDA: Food and Drug Administration
HR: homologous recombination
HTS: high‐throughput screening
KRAB: Krüppel‐associated box
NHEJ: non‐homologous end‐joining
PAM: protospacer adjacent motif
PROTAC: proteolysis‐targeting chimeras
SAM: synergistic activation mediator
sgRNA: single‐guide RNA
SNV: single nucleotide variant
UGI: uracil DNA glycosylase inhibitor

## 1 Introduction

**Fig. 1:**
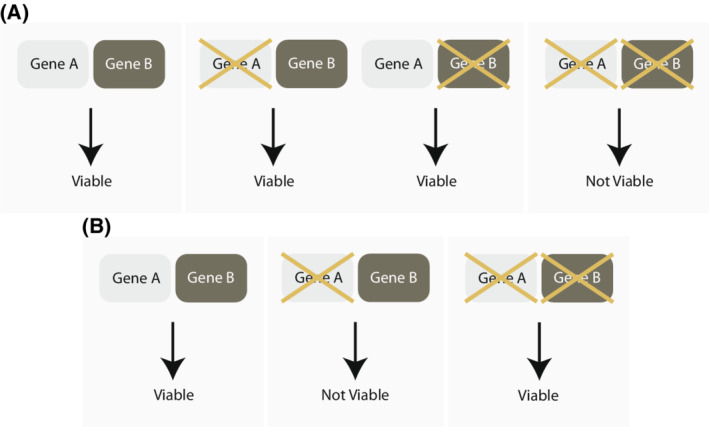
Synthetic lethality and viability. (A) a schematic representation of synthetic lethality. A cell can tolerate the loss of gene a or gene B but cannot survive with the loss of both genes. (B) a schematic representation of synthetic viability. A cell cannot survive the loss of gene a. however, the loss of both genes a and B rescues cellular viability. [Colour figure can be viewed at wileyonlinelibrary.com]

Synthetic lethality occurs when combined mutations in two genes give rise to cell death while the single mutations do not impact cell survival. Conversely, synthetic viability occurs when phenotypic defects caused by mutations in one gene are alleviated by mutations in another gene. In suitable genetic model systems, like yeast, worms, flies and zebrafish, genetic screens have served as powerful tools to uncover such complex negative and positive genetic interactions, when they are associated with a distinct phenotype. In their formative paper, Lee Hartwell and Stephen Friend proposed that these defined molecular contexts and alterations in cancer could be exploited for new therapies [1]. Within the context of cancer, synthetic lethal interactions can represent precise and personalized approaches to treat cancers that harbor specific mutations while synthetic viable interactions can represent mechanisms of resistance to a specific lethal interaction (Fig. 1).

## 2 Synthetic interactions

Genomic instability is one of the hallmarks of cancer, meaning that cancer cells often have dysregulated DNA repair pathways [2]. The increased dependency on compensatory DNA repair pathways represents a vulnerability that can be targeted to specifically kill cancer cells. The concept of synthetic lethality within the DNA damage response (DDR) can be illustrated through the interaction between BRCA1/2 and PARP [3, 4]. Here, mutations within the *BRCA1/2* genes give rise to genomic instability due to defects in homologous recombination (HR) and enhanced replication stress. Members of the PARP family, and specifically PARP1, signal DNA single‐strand breaks thus activating the repair of these lesions. More recently, PARP1 has also been implicated in the repair of Okazaki fragments that occur during discontinuous DNA replication, since these fragments in essence resemble DNA single‐strand breaks [5]. By removing the functions of both BRCA1/2 and PARP, cells accumulate elevated levels of DNA damage, leading to cell death.

Intriguingly, the synthetic lethal interaction between BRCA1/2 and PARP can be overcome through loss of the non‐homologous end‐joining (NHEJ) factor 53BP1 [6]. Loss of 53BP1 promotes processing of broken DNA ends to make them compatible substrates for HR, even in the absence of BRCA1/2. Thus, 53BP1 and BRCA1/2 are important factors that regulate whether DNA double‐strand breaks will be repaired by NHEJ or HR. Similarly, loss of the Shieldin complex renders BRCA1‐deficient cells resistant to PARP inhibition due to its function in promoting end‐joining by restricting resection of DNA double‐strand breaks and subsequent processing by HR [7, 8, 9]. These resistance mechanisms give an insight into the competition between HR and NHEJ components at DNA double‐stranded break sites. Other resistance mechanisms, such as PTIP deficiency in BRCA2‐deficient cells, provide information about replication fork protection. Instead of restoring HR, loss of PTIP inhibits the recruitment of the MRE11 nuclease thus protecting nascent DNA strands from excessive degradation [10].

## 3 CRISPR‐based screens in drug discovery

**Fig. 2:**
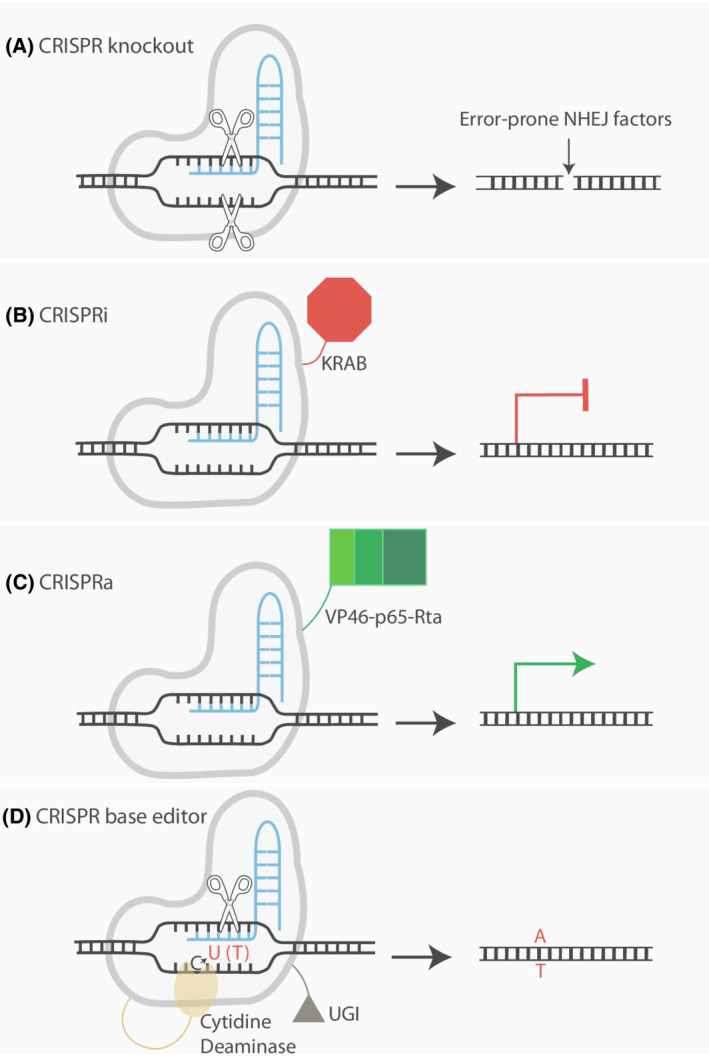
An overview of CRISPR‐Cas9 constructs that are utilized in pooled screens. (A) CRISPR knockout induces a blunt‐ended DNA double‐strand break in a gene of interest. This is then typically repaired by error‐prone non‐homologous end joining (NHEJ) resulting in a frame‐shift mutation. (B) CRISPR interference (CRISPRi) consists of a dCas9 fused with a Krüppel associated box (KRAB) domain which transcriptionally silences the promoter of interest. (C) CRISPR activation (CRISPRa) also consists of a dCas9 which is fused with a VP46 and the activator domains of the transcription factors p65 and Rta. This transcriptionally activates the promoter of interest. (D) Base editor, here presented by a cytidine base editor, allows introduction of single nucleotide variants. The C base is converted into a U base intermediate. The fused uracil DNA glycosylase inhibitor (UGI) domain protects the newly formed uracil intermediate from uracil‐DNA glycosylase. The nickase Cas9 (nCas9) generates a nick on the sgRNA target strand thereby activating mismatch repair (MMR) to excise the nicked target strand base. This converts the original U:G mismatch into a U:A pair. The U is finally converted to a T base via repair or replication. [Colour figure can be viewed at wileyonlinelibrary.com]

The identification and use of CRISPR‐Cas9 for gene editing coupled with genome‐wide libraries for knock‐out, knock‐down, overexpression and base editing of genes has fuelled our understanding of genetic interactions (Fig. 2). Moreover, the advent of induced pluripotent cells, organoid systems and CRISPR‐Cas9 gene editing to recapitulate the precise mutations present in patients have enabled the exploration of genetic space responsible for specific phenotypes.

### 3.1 CRISPR knockout

To initiate editing, the Cas9‐single guide RNA (sgRNA) duplex unwinds DNA and searches for complementarity in the 20 base‐pair region upstream to the Protospacer Adjacent Motif (PAM). Cas9 then cleaves the DNA generating a blunt‐ended DNA double‐stranded break. Remarkably, this breakage is an ATP and GTP independent process and instead harnesses the binding interactions between the Cas9 and the PAM [11]. The main pathway of choice following a DNA double‐stranded break is the error prone NHEJ, which results in small insertions and deletions at the site of the break, typically producing a frame‐shift mutation [12].

**Fig. 3:**
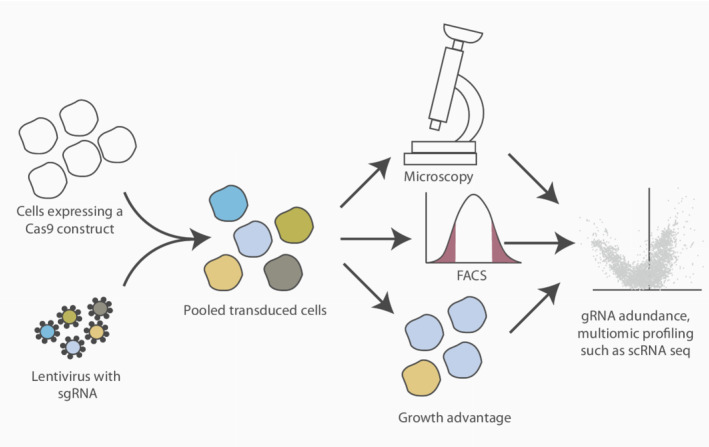
An example of a pooled CRISPR screen. Cells expressing a Cas9 construct are transduced with a lentivirus sgRNA library in bulk. Following perturbation and selection, the cells are then exposed to a survival challenge (e.g. exposure to a compound). Alternatively, the population can be phenotypically characterized via microscopy or fluorescence‐activated cell sorting (FACS) or analyzing the growth advantage in the population. Finally, sgRNA abundance can be determined by next‐generation sequencing. Multi‐omic profiling such as single‐cell RNA‐sequencing (scRNA‐seq) can also be utilized. [Colour figure can be viewed at wileyonlinelibrary.com]

Given its scale and high‐throughput nature, CRISPR knockout screens in the DDR field and elsewhere have utilized a pooled format for discovery where multiple targeting constructs are used in parallel (Fig. 3). This allows for a genome wide interrogation of gene–gene and gene–drug interactions. Following the perturbations and survival challenges, sgRNAs are amplified and sequenced to determine their relative abundance for negative or positive selection. An alternative readout to cell survival, or proliferation, is to assess functional, or phenotypic, parameters through the use of fluorescence‐activated cell sorting (FACS)‐based readouts. This is typically done by sorting cells based on their expression for a particular marker. More recently, microscopy has also been utilized as a readout, for example, by measuring γH2AX foci (a marker of DNA damage) coupled with *in situ* sequencing [13]. Arrayed screens, which have a physical separation between each perturbation, are typically used for validation and follow‐up studies.

Using pooled CRISPR‐Cas9 knockout screens, PARP activity has been shown to synergize with more than BRCA1/2 loss, and to be synthetic lethal with 73 genes. These range from other components of the HR machinery, as well as factors of the Fanconi anemia repair pathway, ribonucleotide excision repair, splicing and transcription [14]. Within BRCA1/2 deficiency itself, attractive synthetic interactions have recently been identified via CRISPR screening, including CIP2A. Unlike PARP inhibition which promotes replication‐induced DNA lesions that require HR repair, the CIP2A‐TOPBP1 complex prevents the potentially lethal mis‐segregation of acentric chromosomes [15]. Moreover, the use of DNA damaging agents in CRISPR‐Cas9 knockout screens allows for the compilation of rich maps of the DNA damage network [16]. Another approach to systematically identify gene–gene interactions using the CRISPR‐Cas9 system is with a double knockout system [17]. These synergistic and buffering interaction patterns can serve as a ‘phenotypic signature’ to effectively cluster genes into pathways and complexes.

As CRISPR knockout screens become increasingly routine, databases have been curated to collect and store screening data from different institutions. One example of this is The Cancer Dependency Map (depmap.org/portal). An integration of these data sets allows for more cell lines and other models to be compiled. It also provides more statistical power for cancer specific dependencies, revealing additional biomarkers for gene dependency [18].

### 3.2 CRISPR interference

To date, most CRISPR‐Cas9 screens in the DDR field have utilized gene knockouts as genetic perturbations. However, the CRISPR system has been further engineered with other protein constructs to allow for other effector functions at gene loci. CRISPR interference (CRISPRi) involves a nuclease inactive Cas9, usually abbreviated dCas9, fused with a Krüppel associated box (KRAB) domain that silences gene expression after being targeted to the promoter region. The human genome encodes for over 350‐KRAB domain proteins that differ in their potency with regards to gene repression [19]. Out of the plethora of KRAB domains tested, the ZIM3 KRAB domain has proven to be a reliably strong repressor [20].

An interesting tool to precisely titrate levels of gene expression is the use of guides with mismatches in conjunction with the dCas9‐KRAB construct [21]. Typically, sgRNAs with single mismatches close to the PAM region have attenuated activity compared to sgRNA mismatches in the PAM distal region. Other complex bio‐physical interactions also dictate the mismatch guide activity which can been modeled using neural network predictions. Taken together, the approach of tittering gene expression could be useful especially for the DNA damage community as many DDR genes are essential, such as ATR [22] and RAD51 [23].

Unlike Cas9‐mediated knockout, since CRISPRi does not induce a DNA double‐strand break it does not elicit the DDR thus potentially altering the cell's state. CRISPRi (as well as other CRISPR‐based approaches) have been used in concurrent single cell approaches that determine the sgRNA and a high content transcriptional read‐out. Several techniques have been developed using this approach including CROP‐seq [24], Perturb‐seq [25], CRISP‐seq [26] and Mosaic‐seq [27]. This adds a further layer of complexity allowing for a greater understanding of the gene expression landscape and signalling pathway activities following the loss of function of a specified gene. Recently, novel regulators in chromosomal instability have been identified using Perturb‐seq [28]. This technology has also been used to achieve transcriptomic read‐outs with mismatch guide mediated perturbations of essential genes [21].

An important limitation to CRISPRi is the requirement for continuous expression of the dCas9‐KRAB construct and the sgRNA. One technique that has overcome this obstacle is the CRISPRoff system [29] that consists of a dCas9 construct that can elicit DNA methylation and repressive histone modifications for epigenetic silencing. Transient expression of the CRISPRoff system is sufficient to target a broad range of different promoters for long‐term gene repression. However, with this tool it is mostly likely not possible to obtain the different levels of gene inhibition that can be achieved with mismatched sgRNA CRISPRi.

### 3.3 CRISPR activation

CRISPR activation (CRISPRa), similarly to CRISPRi, targets the promoters of loci to induce an epigenetic change. Early CRISPRa constructs consisted of dCas9 fused to the transcriptional activator VP64 which has only very modest effects on transcriptional activation. Currently, one of most used is the synergistic activation mediator (SAM), which recruits the HSF1 and p65 transcriptional activators [30]. Other constructs include the dCas9‐SunTag system [31], which recruits many copies of VP64 and the dCas9–VPR system including activator domains of the transcription factors p65 and Rta [32]. Further studies are required to assess whether the use of a mismatch gRNA in combination with CRISPRa could produce varying levels of gene activation, which may be interesting to explore.

CRISPRa libraries have been utilized to identify interactions for both PARP and ATR inhibitors. Using a genome wide CRISPRa library, overexpression of ABCB1, encoding for a multidrug resistance protein, was found to confer resistance to PARP inhibition [33]. Interestingly, acquired resistance to olaparib in ovarian cancer cell lines has been reported to occur via upregulation of MDR‐1, the protein encoded by ABCB1 [34]. Using ATR inhibitors and a genome scale CRISPRa library in two human cell lines (MCF10A and HeLa), different genes were found to give rise to resistance in the two cell lines [35]. This would suggest that resistance to ATR inhibitors occurs via different mechanisms in HeLa compared to MCF10A cells.

The overexpression of oncogenes has been shown to increase cellular sensitivity to several DDR inhibitors. For example, high MYC expression, correlating to higher levels of endogenous replication stress, has been shown to sensitize lymphoma cell lines to ATR or WEE1 inhibition [36]. The synthetic lethal relationship between MYC and ATR has been demonstrated in an *in vivo* setting [37]. Overexpression of the cell cycle regulator CCNE1 is synthetic lethal with the inhibition of PKMYT1 kinase [38]. Thus, we propose that CRISPRa‐based genetic screens could potentially be useful in identifying biomarkers of sensitivity and resistance to drugs that inhibit the DDR.

### 3.4 Base editors

Base editors are an innovative addition to the CRISPR‐Cas9 toolbox. Unlike CRISPR knockout, inhibition and activation which provide ‘gene‐level’ information, base editor technology allows for collecting ‘amino acid level’ information by modifying a nucleobase to incorporate a single nucleotide variant (SNV). Many different base editors have been engineered and at present, they can be divided into two classes: cytosine base editors (CBEs) and adenine base editors (ABEs) [39, 40]. Along with a base modification enzyme, CRISPR base editors often employ a Cas9 nickase which produces a single nucleotide cut in the non‐edited DNA strand to trigger repair. CBEs additionally typically have a fused uracil DNA glycosylase inhibitor (UGI) domain, which protects the newly formed uracil intermediate from uracil‐DNA glycosylase [41]. In total, CBEs and ABEs can efficiently elicit four transition mutations in cytosine (C), thymine (T), adenine (A) and guanine (G) as follows: C → T, G → A, A → G, T → C). However, this approach cannot currently perform the eight transversion mutations (C → A/G, G → C/T, A → C/T, T → A/G) [39].

There are over 75 000 known pathogenic gene variants [42]. These come in a variety of distinct categories ranging from single point mutations, duplications, copy number changes, insertions and deletions, among others. Base editors allow for the correction of transition point mutations at a target pathogenic site without the requirement of a double‐stranded DNA break. A major challenge for base editing is to prevent‐off target edits. Most base editors can edit with a small window to where the sgRNA is recruited to. Therefore, the gene sequence neighboring the targets of interest should be considered for gRNA design. Base editors with narrower editing windows [43, 44] and more flexible PAMs [45, 46] have now been developed. To predict base editing outcomes and ‘bystander’ edits, machine learning models have now been developed [47].

By utilizing base editors in a pooled screen format, more can be learnt about SNVs in DDR. Recently, different nucleotide variants of *TP53* have been ranked based on their loss of tumor suppression [48]. Novel variants in ATM kinase that promote genome instability have been described and mutations in *CHK2* that were variants of unknown significance for cancer can be recategorized as loss of function mutations [49]. Treating pooled perturbations with DNA damaging agents allows for a greater understanding of variants that can change drug sensitivity [50]. Overall, base editors will be a useful asset to further our understanding of how SNVs underpin DDR related diseases, cancer predisposition and sensitivity to DDR inhibitors and DNA damaging agents.

## 4 Advancements in chemical compounds

In addition to exploring genetic space, chemical space can also be explored in cancer therapy. High‐throughput screening (HTS) has enabled automation and miniaturized bioassays; thus, a large number of candidate compounds or genetic modulators can be assessed for a specific biomolecular activity. In addition, HTS can also support improved understanding of biochemical processes and as such has thus become a powerful discovery tool that relies on suitable cellular systems, which can reflect the disease mechanism. Coupled with advances in developing chemical compounds, HTS has led to improvements in drug discovery, ranging from synthesis and mode of action, all the way to drug metabolism and side effects.

### 4.1 Small molecule inhibitors for the DDR

#### 4.1.1 PARP inhibitors

PARP inhibitors are the first and best characterized targeted therapy utilizing synthetic lethality in the DDR. To date four PARP inhibitors have been approved by the Food and Drug Administration (FDA): talazoparib, rucaparib, niraparib and olaparib all of which are NAD^+^ analogs that compete with NAD^+^ for binding of the PARP‐1 catalytic domain [51]. When a given PARP inhibitor binds to PARP, firstly, PARP can no longer PARylate other proteins involved in the repair process. Second, the completion of base excision repair (BER) and DNA single‐strand break repair (SSB) requires the dissociation of PARP from the DNA. Consequently, inhibition of auto‐PARylation results in PARP ‘trapping’ on DNA. When a replication fork approaches, DNA SSBs with bound PARP are converted to DSBs which cannot be processed in HR‐deficient cells [52]. PARP inhibitors vary in their trapping abilities which correlates with cytotoxicity [53]. Talazoparib has the greatest PARP trapping capability out of the four FDA approved drugs [54]. Each of the PARP inhibitors approved, whilst largely target PARP1, has unique off‐target effects with other members of the PARP family and has varying pharmacological profiles across the kinome, the set of protein kinases encoded by the genome [55].

With the implementation of pooled CRISPR screens, BER intermediates, including FEN1 and APEX2, have been identified to enhance sensitivity of PARP inhibition in HR‐deficient backgrounds [56, 57]. This is proposed to be because abasic sites and DNA SSBs accumulate in BER‐deficient backgrounds and provide a source for PARP trapping. Another important determinant of PARP sensitivity identified in screens is the chromatin remodeler ALC1/CHD1L [58]. In the absence of ALC1, PARP is retained on chromatin further enhancing PARP trapping.

Despite good initial response rates with PARP inhibitors, many cancers eventually develop resistance. Numerous mechanisms of resistance have been noted in preclinical studies including the full or partial restoration of BRCA [59, 60], restoration of HR in BRCA1 deficient cells [61, 62] fork protection in BRCA2‐deficient cells [63] and rescues in both PARP trapping [64] and PARylation activity [65]. Inhibitors of poly(ADP‐ribose) glycohydrolase (PARG) have generated interest as a mechanism of overcoming PARylation signaling‐based resistance [66, 67].

The development of a particular resistance mechanism can potentially confer vulnerabilities in other DNA repair pathways and thus provide targets for second‐line therapies. For example, BRCA1‐deficient cells that lose Shieldin/53BP1 to restore HR become hypersensitive to ionizing radiation and cisplatin [7]. Additionally, BRCA1‐deficient cells are hypersensitive to the depletion of nucleotide sanitizers such as DNPH1, resulting in the incorporation of the aberrant nucleotide hydroxyuridine [68]. Taken together, understanding which PARP inhibitors should be used to treat a particular cohort, the development of more selective PARP inhibitors, investigating PARP beyond BRCA1/2, and knowledge on resistance mechanisms in the clinical setting will be important for improving clinical outcomes.

#### 4.1.2 ATM, ATR and DNA PKcs inhibitors

ATM, ATR and DNA PKcs, previously described as the trinity at the heart of the DNA damage response [69], are attractive targets for cancer therapeutics. ATR, despite being an essential kinase, has therapeutic relevance and several inhibitors have been synthesized and tested in clinical trials. These include berzosertib (also known as M6620 or VX‐970), ceralasertib (AZD6738), BAY‐1895344 and more recently M4344. Genome wide interrogations using CRISPR knockout have highlighted potential biomarkers for resistance including the loss of CDC25A [70] and loss of cyclin C and CDK8 [71] to ATR inhibition.

The kinase ATM, an important protein in signaling DNA DSBs, has three inhibitors that are currently tested in clinical trials. These include AZD0156, AZD1390 and M3541. AZD1390 has particularly good blood–brain barrier permeability profiles [72] and is being tested in clinical trials for gliomas (NCT05182905). Both AZD1390 and M3541 are being tested in combination with radiotherapy. Inhibiting ATM kinase activity has been shown to sensitize cells to radiation [73] also supported by the sensitivity of Ataxia–telangiectasia patients to radiation [74].

DNA‐PKcs is a key component in NHEJ and similarly to ATM, inhibition of this kinase sensitizes cells to radiation and synergizes with topoisomerase inhibitors [75]. Various DNA‐PKcs inhibitors that have entered clinical trials including CC‐115, M3814 (nedisertib or peposertib) and AZD7648. An important recent development was the first cryo‐electron microscopy (EM) images of DNA‐PKcs bound to various inhibitors and ATP analogs [76]. Like many kinase targets, the current drug candidates have been developed from high‐throughput screening targeting the ATP‐binding site. However, molecular details of the modes of action of such candidates have been unclear. This is particularly the case for DNA‐PKcs given its large size. From the cryo‐EM images generated, precise inhibitor binding can be elucidated as well as the effect this has on DNA‐PKcs dimerization and its higher order structures.

#### 4.1.3 Checkpoint and cell cycle inhibitors

Targets of CHK1 and CHK2, downstream of ATR and ATM, are important kinases involved in coordinating the DNA damage response and cell cycle arrest. Several compounds have been tested in clinical trials, many of which have shown low to modest anti‐tumor effects alongside toxicity, including AZD7762 [77] and GDC‐0575 [78]. Prexasertib (LY2606368), an ATP competitive inhibitor of CHK1, has shown promising potency in recent trials [79]. Further trials with this inhibitor are currently ongoing (NCT04095221, NCT04023669, NCT02649764).

The WEE1 kinase phosphorylates and inhibits CDK1 and CDK2, thereby exerting control over the intra‐S and G2‐M checkpoints [80, 81]. Recently, WEE1 has been described to protect against fork degradation at stalled replication forks [82]. Cells with higher levels of endogenous replication stress, for instance promoted by high expression of MYC and cyclin E [36], were shown to be hypersensitive to WEE1 inhibition. Loss of WEE1 activity is synthetic lethal with diminished histone H3K36me3, and thus cancers with low levels of H3K36me3 benefit from WEE1 inhibition [83]. This is due to a reduction of RRM2, a ribonucleotide reductase subunit, leading to a depletion in the nucleotide pool. This potential biomarker is being tested in a phase 2 clinical trial on advanced solid tumors (NCT03284385). AstraZeneca's WEE1 inhibitor, AZD1775, has been tested on a wide range of tumors and has shown promising results in a uterine carcinosarcoma study [84]. Currently, there are 26 clinical trials that are recruiting or are active in order to test AZD1775 in human subjects (clinicaltirals.gov).

PLK1 is also a kinase involved in the G2‐M checkpoint and overexpressed in many cancers [85]. Volasertib is the most advanced PLK1 inhibitor in clinical development with recent phase 2 and 3 clinical trials in acute myeloid leukemia, among others. In combination with chemotherapies, volasertib has had variable response rates [86, 87]. An orally available ATP competitive inhibitor of PLK1 is currently undergoing clinical trials including in colorectal cancer with KRAS mutations (NCT03829410). Like with many of the DDR inhibitors, biomarkers for sensitivity are in short supply. A recent CRISPR knockout screen has highlighted a potential non‐canonical cell cycle gene, ARID1A, that when lost promoted sensitivity to PLK1 inhibition [88]. Additionally, high PRC1 expression, which correlated with poor survival, increased sensitivity to PLK1 inhibition in Ewing sarcoma [89].

#### 4.1.4 New and emerging small‐molecule inhibitors for the DDR

The DNA polymerase POLθ is upregulated in a number of cancers and is associated with a poor prognosis [90, 91]. The polymerase has several described functions involving POLθ‐mediated end joining [92] and translesion polymerase synthesis [93]. Inhibition of POLθ is synthetically lethal with BRCA1/2 deficiency [94]. One hypothesis for this is that the loss of both HR and POLθ‐mediated end joining that provide a synthetic lethal relationship. An alternative hypothesis is that POLθ functions in processing the single‐stranded DNA gaps that occur at replication forks in a BRCA‐deficient background. POLθ inhibition may also provide a therapeutic option to treat BRCA‐deficient cancers that are resistant to PARP inhibition due to mutations in 53BP1/Shieldin [95]. POLθ contains a polymerase and a helicase domain, both of which are druggable targets [96, 97]. Inhibition of either the polymerase domain [95] or the helicase domain [98] of POLθ has been reported to benefit BRCA deficient cancers. Building on these studies, Artios Pharma recently dosed their first patient in a phase 1/2a study (NCT04991480) with a POLθ inhibitor targeting the polymerase domain (https://www.artiospharma.com/2021/09/28/artios‐doses‐first‐patient‐in‐phase‐1‐2a‐study‐of‐pol%CE%B8‐inhibitor‐art4215/). Conversely, novobiocin, an antibiotic first described in the 1950s that inhibits the helicase domain of POLθ, is currently being tested in clinical trials [98]. Additionally, POLθ pre‐clinical programs have been disclosed by various pharmaceutical companies including Ideaya, Breakpoint Therapeutics and Repare Therapeutics.

KSQ pharmaceuticals has announced a first dose in a cancer patient in 2021 with a USP1 inhibitor. A phase 1 clinical trial of KSQ‐4279 is currently recruiting both as a monotherapy and in combination with PARP inhibition in patients with advanced solid tumors (NCT05240898). In addition, other companies, including Tango, have disclosed a USP1 inhibitor program. USP1 is a deubiquitinase implicated in DNA repair. Along with USP1 associated factor 1 (UAF1), it removes monoubiquitin signals from PCNA, FANCI and FANCD2 [99, 100, 101]. USP1 is synthetic lethal with BRCA1‐deficient cell lines and is involved in binding and stabilizing replication forks [102]. Like POLθ inhibition, it may provide a potential avenue for exploiting HR deficiency.

Other companies are also expected to announce their first patient dosing of new drugs including Cyteir Therapeutics' RAD51 inhibitor (NCT03997968), Repare Therapeutics' PKMYT1 inhibitor (NCT05147272) and ATR inhibitor (NCT04497116), Artios' ATR inhibitor (NCT04657068), WEE1 inhibitors from Debiopharm (NCT03968653) and Zentalis (NCT04158336). Inhibitors for the WRN helicase, which had generated a lot of interest due to being synthetically lethal with microsatellite instability [103, 104, 105, 106, 107], are currently in the discovery phase in several companies (https://www.nimbustx.com/pipeline‐targets/, https://www.beactica.com/pipeline, https://www.xposetx.com/pipeline, https://ryvu.com/pipeline/).

### 4.2 Targeted protein degradation

**Fig. 4:**
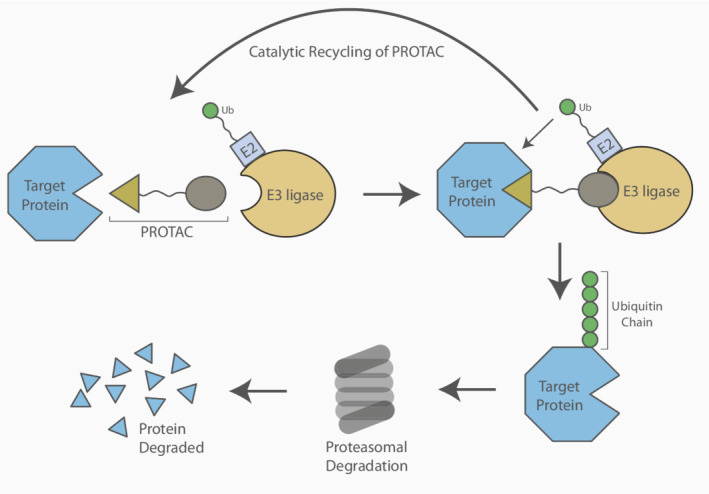
A target protein degraded using a proteolysis targeting chimera (PROTAC). The induced proximity of the target protein and the E3 ligase promotes the polyubiquitination of the target protein. This post‐translational modification signals for its degradation. The PROTAC can be catalytically recycled. [Colour figure can be viewed at wileyonlinelibrary.com]

A significant challenge in ‘occupancy driven’ pharmacology is the observation that many proteins lack a ‘druggable’ pocket in which small molecule inhibitors can bind. An alternative promising strategy is proteolysis‐targeting chimeras (PROTACs). These drugs hijack the ubiquitin proteasome system and promote the degradation of a target protein [108]. This effectively removes all possible functions of the protein as opposed to occupying a catalytic or allosteric domain [109]. PROTACs are bifunctional. They contain a small‐molecule binder, also commonly called a warhead, which attaches to the target protein, and E3 ubiquitin ligase binding domain which are held together by a linker. This induces proximity between the target protein and the E3 ubiquitin ligase, thereby promoting its ubiquitination and degradation (Fig. 4).

Another targeted proteolysis strategy includes molecular glues. These are molecules that stabilize the interaction between two proteins without showing a detectable affinity towards (at least) one of the binding partners. They do not contain a linker and may not directly interact with the protein of interest. Some interact with the E3 ubiquitin ligase, thereby influencing the enzymes interaction interface, resulting in the recruitment of the target protein [110]. Molecular glue discovery is challenging and would require a backwards approach involving screening E3 ligase binding molecules or large protein panels for a desired phenotype.

No DDR PROTACs or molecular glues are currently in clinical trials. PROTACs of PARP have been tested *in vitro* [111]. Recently, a range of PROTACs for WEE1 have been developed [112]. This was achieved using AstraZeneca's AZD1775 as the warhead, E3 ligase binders of VHL and CRBN along with linkers of different lengths and compositions. Recently, CDK12‐cyclin K PROTACs and molecular glues have shown synergistic effects with DNA damage‐inducing drugs [113, 114, 115].

An increasing amount of proteolysis therapeutics are expected to enter clinical development. Chemoproteomic approaches and fragment‐based ligand discovery will accelerate identifying new relevant ligands [116, 117]. Identifying functions for E3 ubiquitin ligases will be important as the majority remain poorly understood. Targeting a given protein for degradation will provide its own unique challenges regarding the emergence of resistance. Modifications in the core component of the E3 ubiquitin ligase is one mechanism by which this can occur [118, 119].

## 5 Future perspectives

The ability to introduce specific mutations using CRISPR‐based approaches represents an exciting opportunity to investigate how alterations in DNA contribute to resistance or cancer development. Here, we have described how base editors can be used to gain mechanistic insights at the single nucleotide level, yet these approaches are limited with regards to the spectrum of mutations that can be introduced. The discovery of prime editing, a genome editing technology, that can introduce a wide range of mutations has the potential to be very useful in studying disease‐relevant mutations [120] yet its reduced efficiency has precluded it from being used in screens. However, much effort is directed towards making prime editing more efficient and thus future CRISPR‐based screens might include prime editors [121, 122].

Here, we have largely discussed the implementation of CRISPR‐based screens in cancer cell lines. Since they better mimic *in vivo* conditions, developments in the establishment and utilization of organoids represent an exciting opportunity in which to perform such screens. Indeed, genome‐scale CRISPR screens have been reported in human intestinal organoids [123] as well as mouse stomach organoids [124] thus highlighting the feasibility of such an approach. Yet, such 3D systems have their limitations. Consequently, researchers are moving towards preforming CRISPR screens *in vivo* to unravel complex signaling networks within their physiological environment [125, 126]. Improvements in the performance of *in vivo* CRISPR screens will lead to a wider use thus allowing genetic space to be explored in more clinically relevant settings.

## Conflict of interest

The authors declare no conflict of interest.

## Author contributions

JIL conceptualized the manuscript with JW. Both JW and JIL contributed equally to writing the manuscript.

## References

[1] Hartwell LH , Szankasi P , Roberts CJ , Murray AW , Friend SH . Integrating genetic approaches into the discovery of anticancer drugs. Science. 1997;278:1064–8. 9353181 10.1126/science.278.5340.1064

[2] Hanahan D , Weinberg RA . Hallmarks of cancer: the next generation. Cell. 2011;144:646–74. 21376230 10.1016/j.cell.2011.02.013

[3] Farmer H , McCabe H , Lord CJ , Tutt AHJ , Johnson DA , Richardson TB , et al. Targeting the DNA repair defect in BRCA mutant cells as a therapeutic strategy. Nature. 2005;434:917–21. 15829967 10.1038/nature03445

[4] Bryant HE , Schultz N , Thomas HD , Parker KM , Flower D , Lopez E , et al. Specific killing of BRCA2‐deficient tumours with inhibitors of poly(ADP‐ribose) polymerase.[erratum appears in nature. 2007 may 17;447(7142):346]. Nature. 2005;434:913–7. 15829966 10.1038/nature03443

[5] Vaitsiankova A , Burdova K , Sobol M , Gautam A , Benada O , Hanzlikova H , et al. PARP inhibition impedes the maturation of nascent DNA strands during DNA replication. Nat Struct Mol Biol. 2022;29:329–38. 35332322 10.1038/s41594-022-00747-1 PMC9010290

[6] Bunting SF , Callén E , Wong N , Chen H , Polato F , Gunn A , et al. 53BP1 inhibits homologous recombination in Brca1‐deficient cells by blocking resection of DNA breaks. Cell. 2011;141:243–54. 10.1016/j.cell.2010.03.012 PMC2857570 20362325

[7] Dev H , Chiang TWW , Lescale C , de Krijger I , Martin AG , Pilger D , et al. Shieldin complex promotes DNA end‐joining and counters homologous recombination in BRCA1‐null cells. Nat Cell Biol. 2018;20:954–65. 30022119 10.1038/s41556-018-0140-1 PMC6145444

[8] Ghezraoui OC , Becker JR , Bilham K , Moralli D , Anzilotti C , Fischer R , et al. 53BP1 cooperation with the REV7‐Shieldin complex underpins DNA structure‐specific NHEJ Shieldin is essential for REV7‐dependent DNA end‐protection and NHEJ during CSR, and supports toxic NHEJ in BRCA1‐deficient cells, yet is dispensable for REV7‐dependent. Nature. 2018;560:122–7. 30046110 10.1038/s41586-018-0362-1 PMC6989217

[9] Noordermeer SM , Adam S , Setiaputra D , Barazas M , Pettitt SJ , Ling AK , et al. The shieldin complex mediates 53BP1‐dependent DNA repair. Nature. 2018;560:117–21. 30022168 10.1038/s41586-018-0340-7 PMC6141009

[10] Ray Chaudhuri A , Callen E , Ding X , Gogola E , Duarte AA , Lee J‐E , et al. Replication fork stability confers chemoresistance in BRCA‐deficient cells. Nature. 2016;535:382–7. 27443740 10.1038/nature18325 PMC4959813

[11] Sternberg SH , Redding S , Jinek M , Greene EC , Doudna JA . DNA interrogation by the CRISPR RNA‐guided endonuclease Cas9. Nature. 2014;507:62–7. 24476820 10.1038/nature13011 PMC4106473

[12] Doench JG . Am i ready for CRISPR? A user's guide to genetic screens. Nat Rev Genet. 2018;19:67–80. 29199283 10.1038/nrg.2017.97

[13] Funk L , Su K‐C , Feldman D , Singh A , Moodie B , Blainey PC & Cheeseman IM (2021) The phenotypic landscape of essential human genes, *bioRxiv*. 2021.11.28.470116. [PREPRINT] 10.1016/j.cell.2022.10.017 PMC10482496 36347254

[14] Zimmermann M , Murina O , Reijns MAM , Agathanggelou A , Challis R , Tarnauskaite Ž , et al. CRISPR screens identify genomic ribonucleotides as a source of PARP‐trapping lesions. Nature. 2018;559:285–9. 29973717 10.1038/s41586-018-0291-z PMC6071917

[15] Adam S , Rossi SE , Moatti N , de Marco ZM , Xue Y , Ng TF , et al. The CIP2A–TOPBP1 axis safeguards chromosome stability and is a synthetic lethal target for BRCA‐mutated cancer. Nat Cancer. 2021;2:1357–71. 35121901 10.1038/s43018-021-00266-w

[16] Olivieri M , Cho T , Álvarez‐Quilón A , Li K , Schellenberg MJ , Zimmermann M , et al. A genetic map of the response to DNA damage in human cells. Cell. 2020;182:481–496.e21. 32649862 10.1016/j.cell.2020.05.040 PMC7384976

[17] Han K , Jeng EE , Hess GT , Morgens DW , Li A , Bassik MC . Synergistic drug combinations for cancer identified in a CRISPR screen for pairwise genetic interactions. Nat Biotechnol. 2017;35:463–74. 28319085 10.1038/nbt.3834 PMC5557292

[18] Pacini C , Dempster JM , Boyle I , Gonçalves E , Najgebauer H , Karakoc E , et al. Integrated cross‐study datasets of genetic dependencies in cancer. Nat Commun. 2021;12:1–14. 33712601 10.1038/s41467-021-21898-7 PMC7955067

[19] Schmitges FW , Radovani E , Najafabadi HS , Barazandeh M , Campitelli LF , Yin Y , et al. Multiparameter functional diversity of human C2H2 zinc finger proteins. Genome Res. 2016;26:1742–52. 27852650 10.1101/gr.209643.116 PMC5131825

[20] Alerasool N , Segal D , Lee H , Taipale M . An efficient KRAB domain for CRISPRi applications in human cells. Nat Methods. 2020;17:1093–6. 33020655 10.1038/s41592-020-0966-x

[21] Jost M , Santos DA , Saunders RA , Horlbeck MA , Hawkins JS , Scaria SM , et al. Titrating gene expression using libraries of systematically attenuated CRISPR guide RNAs. Nat Biotechnol. 2020;38:355–64. 31932729 10.1038/s41587-019-0387-5 PMC7065968

[22] Cimprich KA , Cortez D . ATR: An essential regulator of genome integrity. Nat Rev Mol Cell Biol. 2008;9:616–27. 18594563 10.1038/nrm2450 PMC2663384

[23] Wassing IE , Esashi F . RAD51: beyond the break. Semin Cell Dev Biol. 2021;113:38–46. 32938550 10.1016/j.semcdb.2020.08.010 PMC8082279

[24] Datlinger P , Rendeiro AF , Schmidl C , Krausgruber T , Traxler P , Klughammer J , et al. Pooled CRISPR screening with single‐cell transcriptome readout. Nat Methods. 2017;14:297–301. 28099430 10.1038/nmeth.4177 PMC5334791

[25] Dixit A , Parnas O , Li B , Chen J , Fulco CP , Jerby‐Arnon L , et al. Perturb‐seq: dissecting molecular circuits with scalable single‐cell RNA profiling of pooled genetic screens. Cell. 2016;167:1853–1866.e17. 27984732 10.1016/j.cell.2016.11.038 PMC5181115

[26] Jaitin DA , Weiner A , Yofe I , Lara‐Astiaso D , Keren‐Shaul H , David E , et al. Dissecting immune circuits by linking CRISPR‐pooled screens with single‐cell RNA‐seq. Cell. 2016;167:1883–1896.e15. 27984734 10.1016/j.cell.2016.11.039

[27] Xie S , Duan J , Li B , Zhou P , Hon GC . Multiplexed engineering and analysis of combinatorial enhancer activity in single cells. Mol Cell. 2017;66:285–299.e5. 28416141 10.1016/j.molcel.2017.03.007

[28] Replogle JM , Saunders RA , Pogson AN , Hussmann JA , Lenail A , Guna A , et al. Mapping information‐rich genotype‐phenotype landscapes with genome‐scale Perturb‐seq. Cell. 2022;S0092‐8674(22)00597‐9. 10.1016/j.cell.2022.05.013 Epub ahead of print. PMC9380471 35688146

[29] Nuñez JK , Chen J , Pommier GC , Cogan JZ , Replogle JM , Adriaens C , et al. Genome‐wide programmable transcriptional memory by CRISPR‐based epigenome editing. Cell. 2021;184:2503–2519.e17. 33838111 10.1016/j.cell.2021.03.025 PMC8376083

[30] Konermann S , Brigham MD , Trevino AE , Joung J , Abudayyeh OO , Barcena C , et al. Genome‐scale transcriptional activation by an engineered CRISPR‐Cas9 complex. Nature. 2015;517:583–8. 25494202 10.1038/nature14136 PMC4420636

[31] Tanenbaum ME , Gilbert LA , Qi LS , Weissman JS , Vale RD . A protein‐tagging system for signal amplification in gene expression and fluorescence imaging. Cell. 2014;159:635–46. 25307933 10.1016/j.cell.2014.09.039 PMC4252608

[32] Chavez A , Scheiman J , Vora S , Pruitt BW , Tuttle M , P R Iyer E , et al. Highly efficient Cas9‐mediated transcriptional programming. Nat Methods. 2015;12:326–8. 25730490 10.1038/nmeth.3312 PMC4393883

[33] Clements KE , Schleicher EM , Thakar T , Hale A , Dhoonmoon A , Tolman NJ , et al. Identification of regulators of poly‐ADP‐ribose polymerase inhibitor response through complementary CRISPR knockout and activation screens. Nat Commun. 2020;11:6118. 33257658 10.1038/s41467-020-19961-w PMC7704667

[34] Vaidyanathan A , Sawers L , Gannon A‐L , Chakravarty P , Scott AL , Bray SE , et al. ABCB1 (MDR1) induction defines a common resistance mechanism in paclitaxel‐ and olaparib‐resistant ovarian cancer cells. Br J Cancer. 2016;115:431–41. 27415012 10.1038/bjc.2016.203 PMC4985349

[35] Schleicher EM , Dhoonmoon A , Jackson LM , Clements KE , Stump CL , Nicolae CM , et al. Dual genome‐wide CRISPR knockout and CRISPR activation screens identify mechanisms that regulate the resistance to multiple ATR inhibitors. PLoS Genet. 2020;16:e1009176. 33137164 10.1371/journal.pgen.1009176 PMC7660927

[36] Young LA , O'Connor LO , de Renty C , Veldman‐Jones MH , Dorval T , Wilson Z , et al. Differential activity of ATR and Wee1 inhibitors in a highly sensitive subpopulation of DLBCL linked to replication stress. Cancer Res. 2019;79:3762–75. 31123088 10.1158/0008-5472.CAN-18-2480

[37] Murga M , Campaner S , Lopez‐Contreras AJ , Toledo LI , Soria R , Montaña MF , et al. Exploiting oncogene‐induced replicative stress for the selective killing of Myc‐driven tumors. Nat Struct Mol Biol. 2011;18:1331–5. 22120667 10.1038/nsmb.2189 PMC4894468

[38] Gallo D , Young JT , Fourtounis J , Martino G , Álvarez‐Quilón A , Bernier C , Duffy NM , Papp R , Roulston A , Stocco R , Szychowski J , Veloso A , Alam H , Baruah PS , Bonneau Fortin A , Bowlan J , Chaudhary N , Desjardins J , Dietrich E , Fournier S , Fugère‐Desjardins C , Goullet de Rugy T , Leclaire M‐E , Liu B , Melo H , Nicolas O , Singhania A , Szilard RK , Tkáč J , Yun Yin S , Morris SJ , Zinda M , Gary Marshall C & Durocher D (2021) CCNE1 amplification is synthetic‐lethal with PKMYT1 kinase inhibition. *bioRxiv*. 2021.04.08.438361. [PREPRINT] 10.1038/s41586-022-04638-9 PMC9046089 35444283

[39] Gaudelli NM , Komor AC , Rees HA , Packer MS , Badran AH , Bryson DI , et al. Programmable base editing of T to G C in genomic DNA without DNA cleavage. Nature. 2017;551:464–71. 29160308 10.1038/nature24644 PMC5726555

[40] Komor AC , Zhao KT , Packer MS , Gaudelli NM , Waterbury AL , Koblan LW , et al. Improved base excision repair inhibition and bacteriophage Mu Gam protein yields C:G‐to‐T:A base editors with higher efficiency and product purity. Sci Adv. 2017;3:1–10. 10.1126/sciadv.aao4774 PMC5576876 28875174

[41] Rees HA , Liu DR . Base editing: precision chemistry on the genome and transcriptome of living cells. Nat Rev Genet. 2018;19:770–88. 30323312 10.1038/s41576-018-0059-1 PMC6535181

[42] Landrum MJ , Lee JM , Benson M , Brown G , Chao C , Chitipiralla S , et al. ClinVar: public archive of interpretations of clinically relevant variants. Nucleic Acids Res. 2016;44:D862–8. 26582918 10.1093/nar/gkv1222 PMC4702865

[43] Tan J , Zhang F , Karcher D , Bock R . Engineering of high‐precision base editors for site‐specific single nucleotide replacement. Nat Commun. 2019;10:439. 30683865 10.1038/s41467-018-08034-8 PMC6347625

[44] Kim YB , Komor AC , Levy JM , Packer MS , Zhao KT , Liu DR . Increasing the genome‐targeting scope and precision of base editing with engineered Cas9‐cytidine deaminase fusions. Nat Biotechnol. 2017;35:371–6. 28191901 10.1038/nbt.3803 PMC5388574

[45] Hu JH , Miller SM , Geurts MH , Tang W , Chen L , Sun N , et al. Evolved Cas9 variants with broad PAM compatibility and high DNA specificity. Nature. 2018;556:57–63. 29512652 10.1038/nature26155 PMC5951633

[46] Walton RT , Christie KA , Whittaker MN , Kleinstiver BP . Unconstrained genome targeting with near‐PAMless engineered CRISPR‐Cas9 variants. Science. 2020;368:290–6. 32217751 10.1126/science.aba8853 PMC7297043

[47] Arbab M , Shen MW , Mok B , Wilson C , Matuszek Ż , Cassa CA , et al. Determinants of base editing outcomes from target library analysis and machine learning. Cell. 2020;182:463–480.e30. 32533916 10.1016/j.cell.2020.05.037 PMC7384975

[48] Sánchez‐Rivera FJ , Diaz BJ , Kastenhuber ER , Schmidt H , Katti A , Kennedy M , et al. Base editing sensor libraries for high‐throughput engineering and functional analysis of cancer‐associated single nucleotide variants. Nat Biotechnol. 2022;40:862–73. 35165384 10.1038/s41587-021-01172-3 PMC9232935

[49] Cuella‐Martin R , Hayward SB , Fan X , Chen X , Huang JW , Taglialatela A , et al. Functional interrogation of DNA damage response variants with base editing screens. Cell. 2021;184:1081–1097.e19. 33606978 10.1016/j.cell.2021.01.041 PMC8018281

[50] Hanna RE , Hegde M , Fagre CR , DeWeirdt PC , Sangree AK , Szegletes Z , et al. Massively parallel assessment of human variants with base editor screens. Cell. 2021;184:1064–1080.e20. 33606977 10.1016/j.cell.2021.01.012

[51] Dawicki‐McKenna JM , Langelier MF , DeNizio JE , Riccio AA , Cao CD , Karch KR , et al. PARP‐1 activation requires local unfolding of an autoinhibitory domain. Mol Cell. 2015;60:755–68. 26626480 10.1016/j.molcel.2015.10.013 PMC4712911

[52] Murai J , Huang SYN , Das BB , Renaud A , Zhang Y , Doroshow JH , et al. Trapping of PARP1 and PARP2 by clinical PARP inhibitors. Cancer Res. 2012;72:5588–99. 23118055 10.1158/0008-5472.CAN-12-2753 PMC3528345

[53] Murai J , Pommier Y . PARP trapping beyond homologous recombination and platinum sensitivity in cancers. Annu Rev Cancer Biol. 2019;3:131–50.

[54] Murai J , Huang SYN , Renaud A , Zhang Y , Ji J , Takeda S , et al. Stereospecific PARP trapping by BMN 673 and comparison with olaparib and rucaparib. Mol Cancer Ther. 2014;13:433–43. 24356813 10.1158/1535-7163.MCT-13-0803 PMC3946062

[55] Antolin AA , Ameratunga M , Banerji U , Clarke PA , Workman P , Al‐Lazikani B . The kinase polypharmacology landscape of clinical PARP inhibitors. Sci Rep. 2020;10:1–14. 32066817 10.1038/s41598-020-59074-4 PMC7026418

[56] Álvarez‐Quilón A , Wojtaszek JL , Mathieu M‐C , Patel T , Appel CD , Hustedt N , et al. Endogenous DNA 3′ blocks are vulnerabilities for BRCA1 and BRCA2 deficiency and are reversed by the APE2 nuclease. Mol Cell. 2020;78:1152–1165.e8. 32516598 10.1016/j.molcel.2020.05.021 PMC7340272

[57] Mengwasser KE , Adeyemi RO , Leng Y , Choi MY , Clairmont C , D'Andrea AD , et al. Genetic screens reveal FEN1 and APEX2 as BRCA2 synthetic lethal targets. Mol Cell. 2019;73:885–899.e6. 30686591 10.1016/j.molcel.2018.12.008 PMC6892393

[58] Hewitt G , Borel V , Segura‐Bayona S , Takaki T , Ruis P , Bellelli R , et al. Defective ALC1 nucleosome remodeling confers PARPi sensitization and synthetic lethality with HRD. Mol Cell. 2021;81:767–783.e11. 33333017 10.1016/j.molcel.2020.12.006 PMC7895907

[59] Waks AG , Cohen O , Kochupurakkal B , Kim D , Dunn CE , Buendia Buendia J , et al. Reversion and non‐reversion mechanisms of resistance to PARP inhibitor or platinum chemotherapy in BRCA1/2‐mutant metastatic breast cancer. Ann Oncol. 2020;31:590–8. 32245699 10.1016/j.annonc.2020.02.008 PMC7946408

[60] Drost R , Dhillon KK , van der Gulden H , van der Heijden I , Brandsma I , Cruz C , et al. BRCA1185delAG tumors may acquire therapy resistance through expression of RING‐less BRCA1. J Clin Invest. 2016;126:2903–18. 27454287 10.1172/JCI70196 PMC4966325

[61] Becker JR , Clifford G , Bonnet C , Groth A , Wilson MD , Chapman JR . BARD1 reads H2A lysine 15 ubiquitination to direct homologous recombination. Nature. 2021;596:433–7. 34321663 10.1038/s41586-021-03776-w PMC7619424

[62] Setiaputra D , Durocher D . Shieldin – the protector of DNA ends. EMBO Rep. 2019;20:1–11. 10.15252/embr.201847560 PMC6501030 30948458

[63] Rondinelli B , Gogola E , Yücel H , Duarte AA , van de Ven M , van der Sluijs R , et al. EZH2 promotes degradation of stalled replication forks by recruiting MUS81 through histone H3 trimethylation. Nat Cell Biol. 2017;19:1371–8. 29035360 10.1038/ncb3626

[64] Pettitt SJ , Krastev DB , Brandsma I , Dréan A , Song F , Aleksandrov R , et al. Genome‐wide and high‐density CRISPR‐Cas9 screens identify point mutations in PARP1 causing PARP inhibitor resistance. Nat Commun. 2018;9:1849. 29748565 10.1038/s41467-018-03917-2 PMC5945626

[65] Gogola E , Duarte AA , de Ruiter JR , Wiegant WW , Schmid JA , de Bruijn R , et al. Selective loss of PARG restores PARylation and counteracts PARP inhibitor‐mediated synthetic lethality. Cancer Cell. 2018;33:1078–1093.e12. 29894693 10.1016/j.ccell.2018.05.008

[66] Houl JH , Ye Z , Brosey CA , Balapiti‐Modarage LPF , Namjoshi S , Bacolla A , et al. Selective small molecule PARG inhibitor causes replication fork stalling and cancer cell death. Nat Commun. 2019;10:5654. 31827085 10.1038/s41467-019-13508-4 PMC6906431

[67] Chen S‐H , Yu X . Targeting dePARylation selectively suppresses DNA repair–defective and PARP inhibitor–resistant malignancies. Sci Adv. 2019;5:eaav4340. 30989114 10.1126/sciadv.aav4340 PMC6457938

[68] Fugger K , Bajrami I , Silva Dos Santos M , Young SJ , Kunzelmann S , Kelly G , et al. Targeting the nucleotide salvage factor DNPH1 sensitizes *BRCA* ‐deficient cells to PARP inhibitors. Science. 2021;372:156–65. 33833118 10.1126/science.abb4542 PMC7610649

[69] Blackford AN , Jackson SP . ATM, ATR, and DNA‐PK: the trinity at the heart of the DNA damage response. Mol Cell. 2017;66:801–17. 28622525 10.1016/j.molcel.2017.05.015

[70] Ruiz S , Mayor‐Ruiz C , Lafarga V , Murga M , Vega‐Sendino M , Ortega S , et al. A genome‐wide CRISPR screen identifies CDC25A as a determinant of sensitivity to ATR inhibitors. Mol Cell. 2016;62:307–13. 27067599 10.1016/j.molcel.2016.03.006 PMC5029544

[71] Lloyd RL , Urban V , Muñoz‐Martínez F , Ayestaran I , Thomas JC , de Renty C , et al. Loss of cyclin C or CDK8 provides ATR inhibitor resistance by suppressing transcription‐associated replication stress. Nucleic Acids Res. 2021;49:8665–83. 34329458 10.1093/nar/gkab628 PMC8421211

[72] Durant ST , Zheng L , Wang Y , Chen K , Zhang L , Zhang T , et al. The brain‐penetrant clinical ATM inhibitor AZD1390 radiosensitizes and improves survival of preclinical brain tumor models. Sci Adv. 2018;4:eaat1719. 29938225 10.1126/sciadv.aat1719 PMC6010333

[73] Sarkaria JN , Eshleman JS . ATM as a target for novel radiosensitizers. Semin Radiat Oncol. 2001;11:316–27. 11677656 10.1053/srao.2001.26030

[74] Taylor AMR , Harnden DG , Arlett CF , Harcourt SA , Lehmann AR , Stevens S , et al. Ataxia telangiectasia: a human mutation with abnormal radiation sensitivity. Nature. 1975;258:427–9. 1196376 10.1038/258427a0

[75] Wise HC , Iyer GV , Moore K , Temkin SM , Gordon S , Aghajanian C , et al. Activity of M3814, an Oral DNA‐PK inhibitor, in combination with topoisomerase II inhibitors in ovarian cancer models. Sci Rep. 2019;9:1–7. 31827119 10.1038/s41598-019-54796-6 PMC6906487

[76] Liang S , Thomas SE , Chaplin AK , Hardwick SW , Chirgadze DY , Blundell TL . Structural insights into inhibitor regulation of the DNA repair protein DNA‐PKcs. Nature. 2022;601:643–8. 34987222 10.1038/s41586-021-04274-9 PMC8791830

[77] Sausville E , LoRusso P , Carducci M , Carter J , Quinn MF , Malburg L , et al. Phase I dose‐escalation study of AZD7762, a checkpoint kinase inhibitor, in combination with gemcitabine in US patients with advanced solid tumors. Cancer Chemother Pharmacol. 2014;73:539–49. 24448638 10.1007/s00280-014-2380-5 PMC4486055

[78] Italiano A , Infante JR , Shapiro GI , Moore KN , LoRusso PM , Hamilton E , et al. Phase I study of the checkpoint kinase 1 inhibitor GDC‐0575 in combination with gemcitabine in patients with refractory solid tumors. Ann Oncol. 2018;29:1304–11. 29788155 10.1093/annonc/mdy076

[79] Lee J‐M , Nair J , Zimmer A , Lipkowitz S , Annunziata CM , Merino MJ , et al. Prexasertib, a cell cycle checkpoint kinase 1 and 2 inhibitor, in BRCA wild‐type recurrent high‐grade serous ovarian cancer: a first‐in‐class proof‐of‐concept phase 2 study. Lancet Oncol. 2018;19:207–15. 29361470 10.1016/S1470-2045(18)30009-3 PMC7366122

[80] Tang Z , Coleman TR , Dunphy WG . Two distinct mechanisms for negative regulation of the Wee1 protein kinase. EMBO J. 1993;12:3427–36. 7504624 10.1002/j.1460-2075.1993.tb06017.x PMC413619

[81] Watanabe N , Broome M , Hunter T . Regulation of the human WEE1Hu CDK tyrosine 15‐kinase during the cell cycle. EMBO J. 1995;14:1878–91. 7743995 10.1002/j.1460-2075.1995.tb07180.x PMC398287

[82] Elbæk CR , Petrosius V , Benada J , Erichsen L , Damgaard RB , Sørensen CS . WEE1 kinase protects the stability of stalled DNA replication forks by limiting CDK2 activity. Cell Rep. 2022;38:110261. 35045293 10.1016/j.celrep.2021.110261

[83] Pfister SX , Markkanen E , Jiang Y , Sarkar S , Woodcock M , Orlando G , et al. Inhibiting WEE1 selectively kills histone H3K36me3‐deficient cancers by dNTP starvation. Cancer Cell. 2015;28:557–68. 26602815 10.1016/j.ccell.2015.09.015 PMC4643307

[84] Liu JF , Xiong N , Campos SM , Wright AA , Krasner C , Schumer S , et al. Phase II study of the WEE1 inhibitor Adavosertib in recurrent uterine serous carcinoma. J Clin Oncol. 2021;39:1531–9. 33705205 10.1200/JCO.20.03167

[85] Liu Z , Sun Q , Wang X . PLK1, a potential target for cancer therapy. Transl Oncol. 2017;10:22–32. 27888710 10.1016/j.tranon.2016.10.003 PMC5124362

[86] Döhner H , Lübbert M , Fiedler W , Fouillard L , Haaland A , Brandwein JM , et al. Randomized, phase 2 trial of low‐dose cytarabine with or without volasertib in AML patients not suitable for induction therapy. Blood. 2014;124:1426–33. 25006120 10.1182/blood-2014-03-560557 PMC4148765

[87] Ellis PM , Leighl NB , Hirsh V , Reaume MN , Blais N , Wierzbicki R , et al. A randomized, open‐label phase II trial of volasertib as monotherapy and in combination with standard‐dose pemetrexed compared with pemetrexed monotherapy in second‐line treatment for non–small‐cell lung cancer. Clin Lung Cancer. 2015;16:457–65. 26100229 10.1016/j.cllc.2015.05.010

[88] Srinivas US , Tay NSC , Jaynes P , Anbuselvan A , Ramachandran GK , Wardyn JD , et al. PLK1 inhibition selectively induces apoptosis in ARID1A deficient cells through uncoupling of oxygen consumption from ATP production. Oncogene. 2022;41:1986–2002. 35236967 10.1038/s41388-022-02219-8

[89] Li J , Ohmura S , Marchetto A , Orth MF , Imle R , Dallmayer M , et al. Therapeutic targeting of the PLK1‐PRC1‐axis triggers cell death in genomically silent childhood cancer. Nat Commun. 2021;12:1–12. 34531368 10.1038/s41467-021-25553-z PMC8445938

[90] Kawamura K , Bahar R , Seimiya M , Chiyo M , Wada A , Okada S , et al. DNA polymerase θ is preferentially expressed in lymphoid tissues and upregulated in human cancers. Int J Cancer. 2004;109:9–16. 14735462 10.1002/ijc.11666

[91] Lemée F , Bergoglio V , Fernandez‐Vidal A , Machado‐Silva A , Pillaire MJ , Bieth A , et al. DNA polymerase θ up‐regulation is associated with poor survival in breast cancer, perturbs DNA replication, and promotes genetic instability. Proc Natl Acad Sci USA. 2010;107:13390–5. 20624954 10.1073/pnas.0910759107 PMC2922118

[92] Koole W , van Schendel R , Karambelas AE , van Heteren JT , Okihara KL , Tijsterman M . A polymerase theta‐dependent repair pathway suppresses extensive genomic instability at endogenous G4 DNA sites. Nat Commun. 2014;5:3216. 24496117 10.1038/ncomms4216

[93] Yoon JH , McArthur MJ , Park J , Basu D , Wakamiya M , Prakash L , et al. Error‐prone replication through UV lesions by DNA polymerase θ protects against skin cancers. Cell. 2019;176:1295–1309.e15. 30773314 10.1016/j.cell.2019.01.023 PMC6453116

[94] Ceccaldi R , Liu JC , Amunugama R , Hajdu I , Primack B , Petalcorin MIR , et al. Homologous‐recombination‐deficient tumours are dependent on Polθ ‐mediated repair. Nature. 2015;518:258–62. 25642963 10.1038/nature14184 PMC4415602

[95] Zatreanu D , Robinson HMR , Alkhatib O , Boursier M , Finch H , Geo L , et al. Polθ inhibitors elicit BRCA‐gene synthetic lethality and target PARP inhibitor resistance. Nat Commun. 2021;12:3636. 34140467 10.1038/s41467-021-23463-8 PMC8211653

[96] Zahn KE , Averill AM , Aller P , Wood RD , Doublié S . Human DNA polymerase θ grasps the primer terminus to mediate DNA repair. Nat Struct Mol Biol. 2015;22:304–11. 25775267 10.1038/nsmb.2993 PMC4385486

[97] Newman JA , Cooper CDO , Aitkenhead H , Gileadi O . Structure of the helicase domain of DNA polymerase theta reveals a possible role in the microhomology‐mediated end‐joining pathway. Structure. 2015;23:2319–30. 26636256 10.1016/j.str.2015.10.014 PMC4671958

[98] Zhou J , Gelot C , Pantelidou C , Li A , Yücel H , Davis RE , et al. A first‐in‐class polymerase theta inhibitor selectively targets homologous‐recombination‐deficient tumors. Nat Cancer. 2021;2:598–610. 34179826 10.1038/s43018-021-00203-x PMC8224818

[99] Huang TT , Nijman SMB , Mirchandani KD , Galardy PJ , Cohn MA , Haas W , et al. Regulation of monoubiquitinated PCNA by DUB autocleavage. Nat Cell Biol. 2006;8:339–47. 16531995 10.1038/ncb1378

[100] Sims AE , Spiteri E , Sims RJ , Arita AG , Lach FP , Landers T , et al. FANCI is a second monoubiquitinated member of the Fanconi anemia pathway. Nat Struct Mol Biol. 2007;14:564–7. 17460694 10.1038/nsmb1252

[101] Smogorzewska A , Matsuoka S , Vinciguerra P , McDonald ER , Hurov KE , Luo J , et al. Identification of the FANCI protein, a monoubiquitinated FANCD2 paralog required for DNA repair. Cell. 2007;129:289–301. 17412408 10.1016/j.cell.2007.03.009 PMC2175179

[102] Lim KS , Li H , Roberts EA , Gaudiano EF , Clairmont C , Sambel LA , et al. USP1 is required for replication fork protection in BRCA1‐deficient tumors. Mol Cell. 2018;72:925–941.e4. 30576655 10.1016/j.molcel.2018.10.045 PMC6390489

[103] Chan EM , Shibue T , McFarland JM , Gaeta B , Ghandi M , Dumont N , et al. WRN helicase is a synthetic lethal target in microsatellite unstable cancers. Nature. 2019;568:551–6. 30971823 10.1038/s41586-019-1102-x PMC6580861

[104] Picco G , Cattaneo CM , van Vliet EJ , Crisafulli G , Rospo G , Consonni S , et al. Werner helicase is a synthetic‐lethal vulnerability in mismatch repair– deficient colorectal cancer refractory to targeted therapies, chemotherapy, and immunotherapy. Cancer Discov. 2021;11:1923–37. 33837064 10.1158/2159-8290.CD-20-1508

[105] Lieb S , Blaha‐Ostermann S , Kamper E , Rippka J , Schwarz C , Ehrenhöfer‐Wölfer K , et al. Werner syndrome helicase is a selective vulnerability of microsatellite instability‐high tumor cells. Elife. 2019;8:e43333. 30910006 10.7554/eLife.43333 PMC6435321

[106] Behan FM , Iorio F , Picco G , Gonçalves E , Beaver CM , Migliardi G , et al. Prioritization of cancer therapeutic targets using CRISPR–Cas9 screens. Nature. 2019;568:511–6. 30971826 10.1038/s41586-019-1103-9

[107] Kategaya L , Perumal SK , Hager JH , Belmont LD . Werner syndrome helicase is required for the survival of cancer cells with microsatellite instability. iScience. 2019;13:488–97. 30898619 10.1016/j.isci.2019.02.006 PMC6441948

[108] Sakamoto KM , Kim KB , Kumagai A , Mercurio F , Crews CM , Deshaies RJ . Protacs: chimeric molecules that target proteins to the Skp1‐Cullin‐F box complex for ubiquitination and degradation. Proc Natl Acad Sci USA. 2001;98:8554–9. 11438690 10.1073/pnas.141230798 PMC37474

[109] Bondeson DP , Smith BE , Burslem GM , Buhimschi AD , Hines J , Jaime‐Figueroa S , et al. Lessons in PROTAC design from selective degradation with a promiscuous warhead. Cell Chem Biol. 2018;25:78–87.e5. 29129718 10.1016/j.chembiol.2017.09.010 PMC5777153

[110] Dong G , Ding Y , He S , Sheng C . Molecular glues for targeted protein degradation: from serendipity to rational discovery. J Med Chem. 2021;64:10606–20. 34319094 10.1021/acs.jmedchem.1c00895

[111] Wang S , Han L , Han J , Li P , Ding Q , Zhang QJ , et al. Uncoupling of PARP1 trapping and inhibition using selective PARP1 degradation. Nat Chem Biol. 2019;15:1223–31. 31659317 10.1038/s41589-019-0379-2 PMC6864272

[112] Aublette MC , Harrison TA , Thorpe EJ , Gadd MS . Selective Wee1 degradation by PROTAC degraders recruiting VHL and CRBN E3 ubiquitin ligases. Bioorg Med Chem Lett. 2022;64:128636. 35231578 10.1016/j.bmcl.2022.128636

[113] Mayor‐Ruiz C , Bauer S , Brand M , Kozicka Z , Siklos M , Imrichova H , et al. Rational discovery of molecular glue degraders via scalable chemical profiling. Nat Chem Biol. 2020;16:1199–207. 32747809 10.1038/s41589-020-0594-x PMC7116640

[114] Niu T , Li K , Jiang L , Zhou Z , Hong J , Chen X , et al. Noncovalent CDK12/13 dual inhibitors‐based PROTACs degrade CDK12‐cyclin K complex and induce synthetic lethality with PARP inhibitor. Eur J Med Chem. 2022;228:114012. 34864331 10.1016/j.ejmech.2021.114012

[115] Jiang B , Gao Y , Che J , Lu W , Kaltheuner IH , Dries R , et al. Discovery and resistance mechanism of a selective CDK12 degrader. Nat Chem Biol. 2021;17:675–83. 33753926 10.1038/s41589-021-00765-y PMC8590456

[116] Veggiani G , Gerpe MCR , Sidhu SS , Zhang W . Emerging drug development technologies targeting ubiquitination for cancer therapeutics. Pharmacol Ther. 2019;199:139–54. 30851297 10.1016/j.pharmthera.2019.03.003 PMC7112620

[117] Jacquemard C , Kellenberger E . A bright future for fragment‐based drug discovery: what does it hold? Expert Opin Drug Discov. 2019;14:413–6. 30793989 10.1080/17460441.2019.1583643

[118] Mayor‐Ruiz C , Jaeger MG , Bauer S , Brand M , Sin C , Hanzl A , et al. Plasticity of the Cullin‐RING ligase repertoire shapes sensitivity to ligand‐induced protein degradation. Mol Cell. 2019;75:849–858.e8. 31442425 10.1016/j.molcel.2019.07.013

[119] Lu G , Weng S , Matyskiela M , Zheng X , Fang W , Wood S , et al. UBE2G1 governs the destruction of cereblon neomorphic substrates. Elife. 2018;7:e40958. 30234487 10.7554/eLife.40958 PMC6185104

[120] Anzalone AV , Randolph PB , Davis JR , Sousa AA , Koblan LW , Levy JM , et al. Search‐and‐replace genome editing without double‐strand breaks or donor DNA. Nature. 2019;576:149–57. 31634902 10.1038/s41586-019-1711-4 PMC6907074

[121] Chen PJ , Hussmann JA , Yan J , Knipping F , Ravisankar P , Chen PF , et al. Enhanced prime editing systems by manipulating cellular determinants of editing outcomes. Cell. 2021;184:5635–5652.e29. 34653350 10.1016/j.cell.2021.09.018 PMC8584034

[122] Ferreira da Silva J , Oliveira GP , Arasa‐Verge EA , Kagiou C , Moretton A , Timelthaler G , et al. Prime editing efficiency and fidelity are enhanced in the absence of mismatch repair. Nat Commun. 2022;13:1–11. 35140211 10.1038/s41467-022-28442-1 PMC8828784

[123] Ringel T , Frey N , Ringnalda F , Janjuha S , Cherkaoui S , Butz S , et al. Genome‐scale CRISPR screening in human intestinal organoids identifies drivers of TGF‐β resistance. Cell Stem Cell. 2020;26:431–440.e8. 32142663 10.1016/j.stem.2020.02.007

[124] Murakami K , Terakado Y , Saito K , Jomen Y , Takeda H , Oshima M , et al. A genome‐scale CRISPR screen reveals factors regulating Wnt‐dependent renewal of mouse gastric epithelial cells. Proc Natl Acad Sci USA. 2021;118:1–12. 10.1073/pnas.2016806118 PMC7848749 33479180

[125] Dai M , Yan G , Wang N , Daliah G , Edick AM , Poulet S , et al. In vivo genome‐wide CRISPR screen reveals breast cancer vulnerabilities and synergistic mTOR/hippo targeted combination therapy. Nat Commun. 2021;12:3055. 34031411 10.1038/s41467-021-23316-4 PMC8144221

[126] Bajaj J , Hamilton M , Shima Y , Chambers K , Spinler K , van Nostrand EL , et al. An in vivo genome‐wide CRISPR screen identifies the RNA‐binding protein Staufen2 as a key regulator of myeloid leukemia. Nat Cancer. 2020;1:410–22. 34109316 10.1038/s43018-020-0054-2 PMC8186448

